# Inter-regional mating compatibility among *Bactrocera dorsalis* populations in Thailand (Diptera,Tephritidae)

**DOI:** 10.3897/zookeys.540.6568

**Published:** 2015-11-26

**Authors:** Suksom Chinvinijkul, Sunyanee Srikachar, Phatchara Kumjing, Weera Kimjong, Weerawan Sukamnouyporn, Nongon Polchaimat

**Affiliations:** 1 Department of Agricultural Extension, Ministry of Agriculture and Cooperatives, Chatuchak, Bangkok. 10900. Thailand; 2 Department of Agriculture, Ministry of Agriculture and Cooperatives, Bangkok. Thailand

**Keywords:** Populations, different geographic origins, mating compatibility, field cages, sterile insect technique

## Abstract

Mating compatibility among recently colonized (wildish) populations of *Bactrocera dorsalis* (Hendel) from different geographic origins in Thailand was assessed through inter-regional mating tests. Outdoor octagonal nylon screen field cages containing single potted mango trees (*Mangifera indica* L.) were used. Sexual compatibility was determined using the index of sexual isolation (ISI), the male relative performance index (MRPI), and the female relative performance index (FRPI). The ISI values indicated that the northern population of *Bactrocera dorsalis* from Chiang Mai province was sexually compatible with the southern population of *Bactrocera dorsalis* (previously *Bactrocera papayae*) from Nakhon Si Thammarat province. The MRPI values showed that the northern males had a slightly higher tendency to mate than southern males, while the FRPI data reflected that females of both origins participated equally in matings. In all combinations there were no differences between homotypic and heterotypic couples in mating latency. Southern males tended to mate first with southern females, followed by northern males mating with northern females, while the latest matings involved heterotypic couples, in particular northern males mating with southern females. Overall, more couples were collected from higher parts of the field cage and the upper tree canopy, while there were no differences between the origins of flies in terms of elevation of couples within the cage. Laboratory assessments of fecundity showed no differences in the average number of eggs resulting from inter-regional crosses. Development of immature stages was also equal in the two hybrid crosses, with no differences found in the number of pupae produced, percentage pupal recovery, and percent adult emergence. The practical implication of this study is that colony of *Bactrocera dorsalis* derived from any northern or southern region of Thailand can potentially be used in sterile insect technique programs against this pest.

## Introduction

Polyphagous fruit fly species (Diptera: Tephritidae) are considered major threats to many countries as a result of their pest status, widespread distribution, invasive ability and potential impact on market access (Stephens et al. 2007). These flies infest a broad range of host plants including fruits and vegetables wherever they occur. In south-east Asia, most pest fruit flies belong to the genus *Bactrocera* Macquart, a very large genus of well over 500 species. Several of the most serious *Bactrocera* pest species are indigenous to Thailand and peninsular Malaysia, and amongst these the most important is *Bactrocera dorsalis* (Hendel) (Clarke et al. 2001, 2005).

Following the taxonomic revision of Drew and Hancock (1994), *Bactrocera dorsalis* was considered to occur in a broad swath across much of Asia, from the Indian subcontinent and Andaman Islands to southern China, Taiwan, and southeast Asia, extending southwards to central/southern Thailand as far south as the Isthmus of Kra on the Thai/Malay Peninsula, which Drew and Hancock considered its southern limit. *Bactrocera papayae* (Drew & Hancock) was considered a new species in 1994 separate from *Bactrocera dorsalis* *s.s.* based on subtle morphological characters and identification relied heavily on their respective geographical distributions to discriminate among them (Drew and Hancock 1994). The distribution of *Bactrocera papayae* began at the Isthmus of Kra and extended south to southern Thailand, Malaysia, Singapore, Kalimantan and eastward into the Indonesian archipelago, the large island groups of Sumatra, Java, and Borneo (Drew and Hancock 1994, CABI/EPPO 1998, Clarke et al. 2005, Steck 2007, David and Ramani 2011, Plant Health). The geographical ranges of the two taxa were thought to abut or overlap on or around the Isthmus of Kra, a recognized biogeographic barrier located on the narrowest portion of the Thai peninsula (Krosch et al. 2013). However, due to the recent synonymization of *Bactrocera invadens*, *Bactrocera papayae*, and *Bactrocera philippinensis* with *Bactrocera dorsalis* by Schutze et al. (2015a), the distribution of *Bactrocera dorsalis* now extends throughout much of sub-Saharan Africa, across the Indian subcontinent to the southeast Asian Indo/Malay Archipelago, and as far east as New Guinea and the Philippines.

In Thailand, *Bactrocera dorsalis*, as defined by Drew and Hancock (1994), was trapped in northern and central parts of the country. It was most abundant in the far north with a unimodal population peak, building up from the start of the monsoon season and peaking around June, while September through January was a distinct low period. *Bactrocera papayae* was restricted to southern Thailand, again with a unimodal population peak, with the peak late in the monsoon season (August/September) and dropping off during the dry season (Clarke et al. 2001). *Bactrocera papayae* was considered the most abundant species in different agro-forested locations and in guava, *Psidium guajava* L., orchards in southern Thailand (Danjuma et al. 2013). Recently, fruit fly records in Thailand have been changed following the synonymization of *Bactrocera papayae* with *Bactrocera dorsalis* (Schutze et al. 2015a): it is now recognized that *Bactrocera dorsalis* occurs in all parts of Thailand.

While Schutze et al. (2015a) have synonymized *Bactrocera papayae* with *Bactrocera dorsalis*, as Thai based agricultural researchers we considered it important to carry out local work on diversity in *Bactrocera dorsalis* populations so as to inform local research and policy decisions. Mating compatibility studies among populations of *Bactrocera dorsalis* from northern Thailand and populations from southern Thailand (previously *Bactrocera papayae*) were needed to assess the sexual compatibility of flies from these different origins. Confirming the compatibility of flies from localities 1,500 km apart would endorse the recent synonymization and allow expansion of pilot SIT campaigns, which are currently applied as part of an integrated area-wide approach, to a wide range of environmental and geographical conditions to suppress this pest in Thailand.

## Materials and methods

### Source of flies

Naturally infested fruits from northern Thailand (Chiang Mai province, 19°27'48.5"N; 98°57'50.3"E and 19°23'13.9"N; 98°57'58.9"E) and southern Thailand (Nakhon Si Thammarat province, 8°18'25.8"N; 99°37'50.3"E) were collected from host plants and brought to the fruit fly mass-rearing facility of the Department of Agricultural Extension (DOAE) in Pathumthani province. There they were placed on sawdust in containers to let the larvae mature and pupate. Larvae and pupae were kept at 25±2 °C, 80–90% RH and a 12: 12 (L: D) photoperiod. Emerged adult flies were provided a standard diet consisting of enzymatic yeast hydrolysate and sugar (1: 3) with water supplied *ad libitum*. Wild flies at least 14 days old were identified at the laboratory of the Department of Agriculture, Bangkok, using external morphological characters to confirm their identity in accordance with their taxonomic descriptions (Drew and Hancock 1994). The *Bactrocera dorsalis* colony from Chiang Mai was obtained from the following fruits: mango (*Mangifera indica* L.), rose apple (*Eugenia javanica* Lam.) and star fruit (*Averrhoa carambola* L.), while the *Bactrocera dorsalis* colony from Nakhon Si Thammarat was obtained from Kluai Leb Mu Nang banana (*Musa sapientum* L.) and guava (*Psidium guajava* L.). The identity of flies was further confirmed by using the additional diagnostic tools of pheromone and genetic analyses.

### Pheromone analysis

Five individual rectal (= pheromone) glands of individually methyl-eugenol fed and non-fed sexually mature wild males from southern Thailand, identified as *Bactrocera dorsalis* using morphological characters, were dissected out and stored in 95% alcohol. Samples were sent to the Laboratory of Entomology and Chemical Ecology, Department of Biology, Faculty of Science, Universiti Putra Malaysia for pheromone analysis. Samples were prepared using sample homogenization and solvent concentration under nitrogen before individual glands were transferred to conical glass vials for GC-MS analyses. The methyl-eugenol metabolites: 2-allyl-4, 5-dimethoxyphenol (DMP) and (*E*)-coniferyl alcohol (CF), were detected in all samples that were fed with methyl-eugenol. No samples were found to have the endogenous compounds present in *Bactrocera carambolae* males such as (3-methylbutyl) acetamide, ethyl benzoate, benzamide, 6-oxo-1-nonanol and 1, 6-nonanediol. These results confirmed that the southern colony of *Bactrocera dorsalis* (previously *Bactrocera papayae*) was not contaminated with *Bactrocera carambolae*, which is restricted to southern Thailand (Clarke et al. 2001).

### Genetic analysis

The rest of the bodies of the *Bactrocera dorsalis* males from southern Thailand from which the rectal glands were removed for pheromone analysis, together with sexually mature complete females, were sent for genetic analysis in groups of five preserved in propylene glycol to the Diagnostics for Biosecurity laboratory at Lincoln University, Christchurch, New Zealand. DNA was extracted using PrepGem, and PCR amplified and sequenced for ITS1 using the PCR primers reported in Boykin et al. (2013). DNA sequences were aligned using Sequencher and a neighbor joining sequence-similarity representation developed with MEGA. Sequences of species-verified reference specimens from both Boykin et al. (2013) (specifically *Bactrocera dorsalis* *s.s.* from Taiwan, the Philippines and Malaysia, and *Bactrocera carambolae* from Suriname) and other in-house samples (specifically *Bactrocera dorsalis* [ex *Bactrocera papayae*] from Malaysia and *Bactrocera carambolae* from Suriname and Indonesia, (Mataram, Lombok)) were included in the alignment. The results further confirmed that the colony from the southern population was consistent with *Bactrocera dorsalis* *s.s.*, as per Boykin et al. (2013).

### Flies maintenance protocol

Sexually mature *Bactrocera dorsalis* from each region were exposed to fresh mature Kluai Nam Wa banana (*Musa sapientum* L.) as an oviposition substrate and larval rearing medium. Bananas with eggs were removed from the rearing cages and placed on sawdust in a ventilated container. Pupae were collected daily and held in 20–25 °C room for maturation. This allowed synchronization of development for the sexual compatibility tests. Eight day-old pupae were manually sifted and transferred to standard quality control Plexiglas cages (30 × 40 × 30 cm) (FAO/IAEA/USDA 2003) with screen mesh windows on two sides and one window on the top for water supply. Standard sugar-yeast diet was provided under low-stress conditions after fly emergence.

### Mating compatibility tests

Wildish *Bactrocera dorsalis* flies (2^nd^ and 3^rd^ generations) from northern (Chiang Mai) and southern (Nakhon Si Thammarat) Thailand were used for mating compatibility studies. Adult flies were sorted by sex within five days of emergence and virgin flies, once sexually mature at 21 and 23 days of age, were selected for the field cage tests. Based on preliminary studies to assure the sexual maturation of wild flies (Orankanok et al. 2013), flies were provided for mating compatibility test at 23 days of age for fertile wild males and at 21 days of age for fertile wild females. Flies were marked by immobilizing them and placing a small dot of acrylic color on each fly’s scutum for both males and females in the early morning of each test day. Colors used were alternated between the two populations. All marked flies for the different replicates were maintained in cylindrical plastic containers, 12.5 cm diameter × 15 cm height, with a triangular mesh on the lid where water-agar and sugar-protein diet was supplied.

Mating compatibility tests between populations from the two regions were performed near the DOAE fruit fly mass-rearing facility. Outdoor octagonal field cages (size 120 cm each side and 220 cm height made from 32 mesh nylon screen), with each cage containing a single potted mango tree (*Mangifera indica* L.) ca. 180 cm in height. These were used since mate choice experiments in large, walk-in field cages containing a host plant have proven useful tools in discriminating among closely related sibling species (Cayol et al. 1999, Petit-Marty et al. 2004, Vera et al. 2006, Orozco et al. 2007, Cáceres et al. 2009, Schutze et al. 2013, Bo et al. 2014) and as reviewed by Juárez et al. (2015); protocols for such trials are now well established and widely applied.

Six replicates of control tests (NxN and SxS) and inter-regional (NxS) mating compatibility test were carried out during December 2012, with the six replicates of the two control combinations completed in one day, and the six replicates of the inter-regional combination completed on another. General procedures followed those outlined in the FAO/IAEA/USDA (2003) manual. As *Bactrocera dorsalis* mates at dusk (Arakaki et al. 1984), for each replicate, 20 males from each of the two populations under study were released into the field cage 1 to 2 hours before sunset (16.00–16.30 hrs) and 30 minutes before the females, to give the males enough time to establish territories and form leks (Prokopy and Hendrichs 1979, Shelly and Kaneshiro 1991), followed by the release of 20 females from each of the same two populations (16.30–17.00 hrs). Only healthy marked flies were released; non-active or dead flies were replaced. Temperature, relative humidity and light intensity were recorded immediately after females were released and then every half hour. The formation of copulating pairs was observed continuously, and five minutes after initiation of a mating, the mating pairs were collected into small vials. For each mating couple, the following data were recorded: copulation start time, copulation location in cage, temperature, relative humidity, light intensity, male and female color. For position within cage, high elevation was defined as the top of the canopy of the tree or the ceiling of the field cage; low elevation was defined as the mid-lower canopy or the side-lower cage wall. The mated flies were not replaced or released back into the cage after separation (Cayol et al. 1999). Experiments concluded when flies became inactive, which occurred after sunset when light intensity dropped to 0–10 lux (Schutze et al. 2015b).

### Data analysis

Sexual compatibility was measured using several indices. The Proportion of Flies Mating (PM) measures the suitability of the flies and the environment for mating and represents the overall mating propensity of the flies: Data are discarded if the proportion of flies mating is less than 20% (FAO/IAEA/USDA 2003). The Index of Sexual Isolation (ISI) takes into account the difference existing between homotypic and heterotypic matings; it ranges from -1 (complete negative assortative mating, that is, all matings are with members of the opposite population) to 0 (complete random mating or equal proportion of the possibilities of mating) to +1 (complete positive assortative mating or total sexual isolation, that is total mating isolation of the two populations and males and females only mated with their respective populations). The Male Relative Performance Index (MRPI) highlights any relative difference between males of both populations in terms of overall mating performance; it ranges from -1 (only males of the reciprocal population mated) to 0 (equal mating performance between males of both populations or males of both populations participated equally in mating) to +1 (only males of one population mated). The Female Relative Performance Index (FRPI) highlights any relative difference between females of both populations in terms of overall mating performance. The range of FRPI is similar to MRPI, but applied to females. The combined application of ISI, MRPI, and FRPI provides a comprehensive measure of mating compatibility, as it demonstrates the degree of isolation between populations or species and the relative participation of the sexes of each population or species (Cayol et al. 1999).

### F1 hybrid fitness

Six replications of ten pairs representing both combinations of crosses of flies between *Bactrocera dorsalis* from northern (N) and southern (S) Thailand were individually assessed for the number of eggs, pupae and F1 adults produced. Eggs of each cross were collected and observed from day 7 until 90 day-old parents. All daily collected eggs of each cross were counted, introduced into semi-ripe bananas and laid on fine sawdust in separately ventilated containers for larval maturation. Pupae were separated and transferred into the cages. Adults of each cross were examined for successful emergence.

## Results

### Mating compatibility tests

Six replicates of field cage mating compatibility studies involving northern *Bactrocera dorsalis* and southern *Bactrocera dorsalis* (ex-*Bactrocera papayae*) were completed. The propensity for mating (PM) values was larger than 0.20 in all replicates, indicating that the conditions under which the tests were run were satisfactory. The mean proportion of flies mating in control tests of NN and SS were 0.57 and 0.33, respectively, and 0.38 for the inter-regional tests (NS).

**Figure 1.**
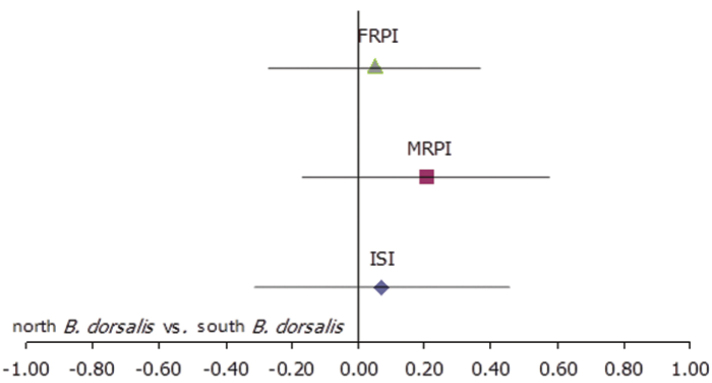
Index of Sexual Isolation (ISI) and Relative Performance Indices for Males (MRPI) and Females (FRPI) for the field cage mating compatibility tests between the two *Bactrocera dorsalis* populations from northern and southern Thailand.

The ISI value of 0.07 illustrates random mating between northern and southern *Bactrocera dorsalis* populations. Northern males showed slightly higher effectiveness at obtaining mates than males of southern population (MRPI = 0.21), while females of both origins participated equally in mating (FRPI = 0.05) (Figure 1).

Total numbers of mated pairs for the control populations of *Bactrocera dorsalis* were lower for the southern population (SS = 80 pairs) and higher for the northern population (NN = 136 pairs). Total pairs across all combinations and comparisons for either homotypic or heterotypic couples of the inter-regional study were not different (SS = 21; NN = 28; SN = 17; NS = 21).

Couples were found mating between 2,000 lux and 35 lux and at temperatures between 28–31 °C. There was no difference in mating latency for couple formation in the two control studies (SS = 67.77 minutes; NN = 70.18 minutes). Averaged across all combinations and comparing homotypic or heterotypic couples of the inter-regional study, there were no differences in mating latency (SS = 69.58 minutes, NN = 70.61 minutes, SN = 71.60 minutes, NS = 72.47 minutes).

More couples in controls and inter-regional mating tests involving the two *Bactrocera dorsalis* populations were collected from high in the field cage (either from the ceiling of the cage or the upper canopy) relative to lower locations; there were no differences between the two population origins in terms of height of the couples inside the field cage (Table 1).

**Table 1.** Mean percentages of pairs of all mating combinations which were collected at two heights within the field cage during mating compatibility tests between populations of *Bactrocera dorsalis* from northern and southern Thailand. High height is defined as the upper canopy of the tree or the ceiling of the field cage; low height is defined as the mid-lower canopy or the mid-lower cage wall.

Crosses		Height	
Male	Female	High (%)	Low (%)
Northern			
*Bactrocera dorsalis*	*Bactrocera dorsalis*	100.00	0.00
Southern			
*Bactrocera dorsalis*	*Bactrocera dorsalis*	97.62	2.38
Northern vs Southern			
(N) *Bactrocera dorsalis*	(N) *Bactrocera dorsalis*	86.57	13.43
(N) *Bactrocera dorsalis*	(S) *Bactrocera dorsalis*	86.11	13.89
(S) *Bactrocera dorsalis*	(N) *Bactrocera dorsalis*	70.83	29.17
(S) *Bactrocera dorsalis*	(S) *Bactrocera dorsalis*	100.00	0.00

### F1 hybrid fitness

Six replicates of ten pairs for each of the two crosses among *Bactrocera dorsalis* from northern and southern Thailand were completed. No differences were found in the mean number of eggs produced, the mean number of pupae produced, percentage pupal recovery (i.e. egg to pupation percentage), and mean percent adult emergence (Table 2).

**Table 2.** Average number of eggs, pupae and adults per female of reciprocal crosses within *Bactrocera dorsalis* populations from northern and southern Thailand.

Crosses		Average number of eggs	Average number of pupae	Average percent of pupae recovery	Average percent emergence of adults	
male	female				Complete	Abnormal
(N) *Bactrocera dorsalis*	(S) *Bactrocera dorsalis*	548.33	119.60	26.33	95.68	4.32
(S) *Bactrocera dorsalis*	(N) *Bactrocera dorsalis*	435.90	144.40	27.44	95.34	4.66

## Discussion

There was no evidence of any pre- or post-mating incompatibility between the *Bactrocera dorsalis* populations from northern (Chiang Mai) and southern (Nakhon Si Thammarat) (ex-*Bactrocera papayae*) Thailand, despite the populations originating from locations approximately 1,500 km apart. The combined data, using the different indices (ISI, MRPI, FRPI), provided a complete and reliable picture of sexual compatibility among northern and southern *Bactrocera dorsalis* populations. The ISI demonstrated good sexual compatibility between northern and southern populations, indicating that individuals from the northern *Bactrocera dorsalis* population mate satisfactorily with those from the southern population. The MRPI indicated a general tendency of wild *Bactrocera dorsalis* males from northern Thailand to succeed in mating in slightly greater proportion with northern or southern females compared to southern males. The FRPI showed that northern and southern females are equally receptive in mating with northern or southern males.

Under similar experimental conditions using field cage mating trials, Schutze et al. 2013 also demonstrated random mating among all pair-wise combinations involving *Bactrocera dorsalis* and *Bactrocera papayae*. At the same time, similar field-cage tests with other fruit flies were able to detect sexual incompatibility among populations of different geographic origin (Vera et al. 2006). All these studies confirm the effectiveness of field cages with trees under semi-natural conditions to evaluate mating compatibility or sexual isolation in cryptic species complexes (Juárez et al. 2015, FAO/IAEA/USDA 2003).

*Bactrocera dorsalis* mates at dusk and the slightly higher proportion of northern males participating in copulations may be a climatic factor at the time of testing in December, when lower temperatures occur at dusk in central Thailand. A potential causal factor for the earlier time at which the southern populations tended to start mating may be the shorter period of optimum light intensity after sunset. Sunsets occurred approximately 14 and 11 minutes of later in Nakhon Si Thammarat relative to Chiang Mai and Bangkok, respectively, and sunset is three minutes earlier in Chiang Mai relative to Bangkok (based on 2012 sunset data; www.sunrise-and-sunset.com). This *ca.* ten minute difference may have affected the mating latency of flies from the extremes of geographical location compared to central Thailand, so that northern flies were delayed while southern flies were enhanced in their mating activity. According to Schutze et al. (2015b) the slight delay in time of sunset at Nakhon Si Thammarat relative to Bangkok may be sufficient to influence mating latency in flies of early-generation laboratory colonies causing earlier mating compared to flies from northern Thailand. Differences in the onset of mating behavior can be readily manipulated by changes in daily light patterns in other tephritid species (Miyatake et al. 2002).

Our inter-regional sexual compatibility results between *Bactrocera dorsalis* and *Bactrocera papayae* (that in the meantime has been synonymized with *Bactrocera dorsalis*) confirmed the high levels of inter-specific mating compatibility among *Bactrocera dorsalis* and *Bactrocera papayae* found in different countries (McInnis et al. 1999, Tan 2000, Wee and Tan 2000, Schutze et al. 2013). Also the laboratory assessments of the viability of the offspring of reciprocal crosses confirmed that *Bactrocera dorsalis* from southern Thailand (ex-*Bactrocera papayae*) and *Bactrocera dorsalis* from northern Thailand represent the same biological species. The capability of females of both populations to produce viable eggs with good pupal recovery after the inter-regional crosses means that there are no post-zygotic barriers to hybrid offspring viability. These results confirmed previous findings by Tan (2003), who demonstrated that *Bactrocera dorsalis* and *Bactrocera papayae* interbreed and produce viable offspring under laboratory conditions. Furthermore, compared to the *Bactrocera dorsalis* mass-reared in our production facility, the level of pupal recovery from all crosses was similar and acceptable.

## Conclusion

The level of sexual compatibility detected in our study confirms the recent synonymization of *Bactrocera papayae* with *Bactrocera dorsalis*. It also opens the possibility of using *Bactrocera dorsalis* flies from either northern or southern populations in Thailand to initiate colonies for mass-rearing facilities. This will allow expanding pilot SIT campaigns, which are currently applied as part of an integrated area-wide approach, to a wider range of environmental and geographical conditions to suppress diverse populations of this pest in Thailand. Also, the mass-reared *Bactrocera dorsalis* flies currently being produced for the ongoing SIT program in Thailand can be used to suppress wild populations of this pest in either northern or southern regions of the country.
